# Silencing of karyopherin α2 inhibits cell growth and survival in human hepatocellular carcinoma

**DOI:** 10.18632/oncotarget.16749

**Published:** 2017-03-31

**Authors:** Yunfeng Yang, Jian Guo, Yuxia Hao, Fuhua Wang, Fengxia Li, Shaomin Shuang, Junping Wang

**Affiliations:** ^1^ College of Chemistry and Chemical Engineering, Shanxi University, Taiyuan, 030006, Shanxi, China; ^2^ Department of Gastroenterology, Shanxi Provincial People's Hospital, Taiyuan, 030012, Shanxi, China; ^3^ Department of General Surgery, Shanxi Provincial People's Hospital, Taiyuan, 030012, Shanxi, China; ^4^ Department of Molecular Biology, Shanxi Cancer Hospital and Institute, Taiyuan, 030013, Shanxi, China

**Keywords:** hepatocellular carcinoma, KPNA2, tumorigenesis, microarray, gene-interaction network

## Abstract

Karyopherin α2 (KPNA2), involved in nucleocytoplasmic transport, has been reported to be upregulated in hepatocellular carcinoma and considered as a biomarker for poor prognosis. However, comprehensive studies of KPNA2 functions in hepatocellular carcinogenesis are still lacking. Our study examine the roles and related molecular mechanisms of KPNA2 in hepatocellular carcinoma development. Results show that KPNA2 knockdown inhibited the proliferation and growth of hepatocellular carcinoma cells *in vitro* and *in vivo*. KPNA2 knockdown also inhibited colony formation ability, induced cell cycle arrest and cellular apoptosis in two hepatocellular carcinoma cell lines, HepG2 and SMMC-7721. Furthermore, gene expression microarray analysis in HepG2 cells with KPNA2 knockdown revealed that critical signaling pathways involved in cell proliferation and survival were deregulated. In conclusion, this study provided systematic evidence that KPNA2 was an essential factor promoting hepatocellular carcinoma and unraveled potential molecular pathways and networks underlying KPNA2-induced hepatocellular carcinogenesis.

## INTRODUCTION

Liver cancer is the fifth most common cancer type and the second leading cause of cancer-associated death globally [1]. In 2012, approximately 782,500 patients were diagnosed with liver cancer, and 745,500 patients died [1]. Among liver cancers, hepatocellular carcinoma (HCC) is the major type and accounts for nearly 90% of primary liver cancer [2]. HCC is highly lethal, and its incidence has tripled in the past thirty years in the United States, while its 5-year survival rate remained below 12% [3]. Currently the standard therapeutic strategies for HCC are liver resection, liver transplantation and ablation, and the 5-year survival rate is higher than 50% with such treatments [4]. However, these treatments are only suitable for a small number of patients diagnosed at an early stage, while most patients are diagnosed with HCC at advanced stages, making HCC high mortality [5]. Though the molecular characteristics of HCC have been explored extensively in recent years with the development of high-throughput sequencing technologies [6, 7], biomarkers, which are essential for HCC early diagnosis, targeted therapies and prognosis, have remained largely elusive. Thus, exploring the molecular pathways underlying HCC tumorigenesis and progression is still in urgent need, as detailed mechanisms will provide insights for the development of more precise diagnosis and more effective and targeted therapies.

Protein transport between the nucleus and the cytoplasm, a process termed nucleocytoplasmic transport, is essential for many different cellular processes, including cell survival and growth, as the localization of proteins is critical to their function [8]. It has been observed frequently that the cellular transport system is deregulated during tumorigenesis and that the distribution of oncogenes and tumor suppressors are disrupted, leading to functional abnormalities and finally tumor initiation and development [9, 10]. Karyopherin α2 (KPNA2), also known as importin α-1 or RAG cohort 1, is a member of the karyopherin α (also called importin α) protein family, which belongs to the karyopherin/importin superfamily involved in nucleocytoplasmic transport through nuclear pore complexes (NPCs) in the nuclear membrane [11, 12]. Thus, it is not unexpected that KPNA2 has been linked to cancer. Elevated KPNA2 expression has been observed in various cancer types, including hepatocellular carcinoma, and its expression level has been positively correlated with diagnosis in hepatocellular carcinoma [13–15]. However, the roles and molecular mechanisms of KPNA2 in hepatocellular carcinogenesis have not been explored in detail.

Here, we chose a lentivirus-mediated short-hairpin RNA (shRNA) strategy to inhibit human KPNA2 expression in two human hepatocellular carcinoma cell lines, HepG2 and SMMC-7721, to explore the impact of KPNA2 on cell growth and survival *in vitro*. It was shown that silencing KPNA2 expression significantly inhibited cell proliferation and colony formation while inducing cell cycle arrest and apoptosis in both hepatocellular carcinoma cell lines. We further revealed that KPNA2 knockdown inhibited hepatocellular tumorigenesis in nude mice. Moreover, global gene expression profiles in HepG2 cells were analyzed using a microarray showed that several key pathways essential for cell growth and survival, such as the cell cycle pathway involved in the G2/M and S phase, ATM signaling, PLK signaling and p53 signaling, were enriched in KPNA2-regulated gene sets. Taken together, our results provide strong evidence to support the causal relationship between KPNA2 and hepatocellular carcinogenesis and provide detailed insights into the KPNA2-mediated molecular pathways/networks underlying KPNA2-associated tumorigenesis.

## RESULTS

### Efficient silencing of KPNA2 expression in human hepatocellular carcinoma cells

**Figure 1:**
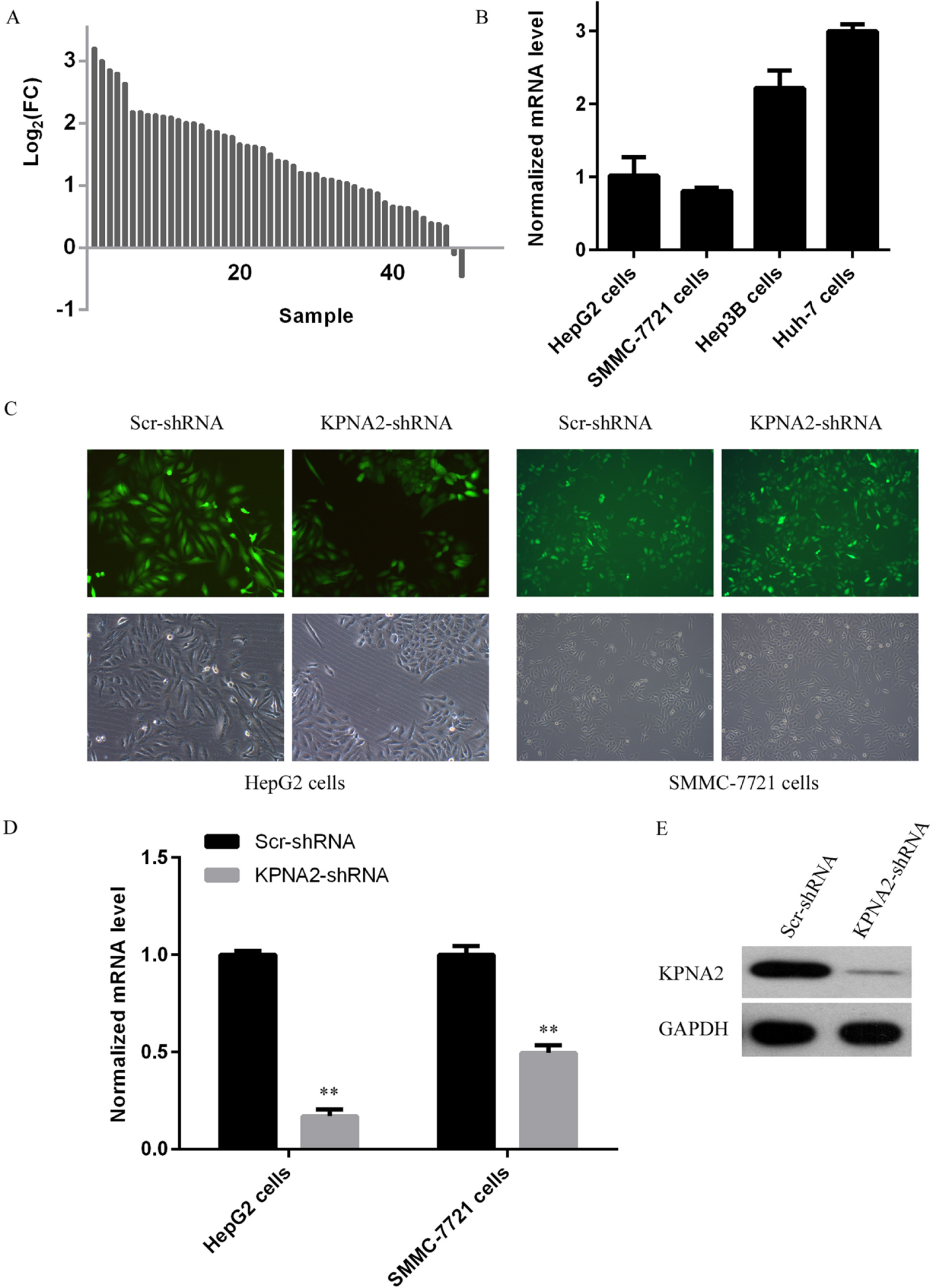
KPNA2 expression status and its knockdown at both the mRNA and the protein levels in human hepatocellular carcinoma cell lines HepG2 and SMMC-7721 using a lentivirus-mediated shRNA strategy (**A**) Line chart for KPNA2 expression at the mRNA level in human hepatocellular carcinoma tissues and adjacent normal tissues obtained from the TCGA database. A total number of 50 paired lung adenocarcinoma samples were used (**B**) KPNA2 expression in four different human hepatocellular carcinoma cell lines, HepG2, SMMC-7721, Hep3B and Huh-7 cells was analyzed with quantitative real-time PCR analysis (normalized to GAPDH mRNA). (**C**) Microscopic images of HepG2 cells and SMMC-7721 cells infected for 48 hours with lentiviruses expressing either Scr-shRNA or KPNA2-shRNA. The upper images were prepared using a fluorescent microscope and show GFP-positive cells; the bottom images were prepared using a light microscope. Magnification: 100 ×. (**D**) The relative KPNA2 mRNA levels in HepG2 cells and SMMC-7721 cells infected with lentiviruses expressing either Scr-shRNA or KPNA2-shRNA was determined by quantitative real-time PCR (normalized to GAPDH mRNA). The data shown here are from one out of three independent experiments (**p < 0.01). (**E**) The relative KPNA2 protein level in 293T cells transfected with a plasmid expressing either Scr-shRNA or KPNA2-shRNA was determined by immunoblotting. The GAPDH protein level was used as an internal control.

Initially, we analyzed KPNA2 expression status in 50 paired hepatocellular carcinoma samples from TCGA database, and showed that KPNA2 expression at the mRNA level was shown to be significantly upregulated in hepatocellular carcinoma tissues as compared to adjacent normal tissues (Figure 1A). KPNA2 expression in four different human hepatocellular carcinoma cell lines, HepG2, SMMC-7721, Hep3B, and Huh-7 cells was further examined using quantitative real-time PCR and it was revealed that Huh-7 cells showed the highest KPNA2 expression, while HepG2 and SMMC-7721 showed similar KPNA2 expression, which were about 1/3 of that in Huh-7 cells (Figure 1B). To investigate the molecular functions of KPNA2 in tumorigenesis of human hepatocellular carcinoma, a lentivirus-mediated short-hairpin RNA (shRNA) strategy was chosen to inhibit KPNA2 expression in two human hepatocellular carcinoma cell lines, HepG2 and SMMC-7721. Lentiviruses expressing either scrambled shRNA (Scr-shRNA) or shRNA specifically targeting human *KPNA2* (KPNA2-shRNA) were prepared and added to HepG2 and SMMC-7721 cells. As the lentivirus contained a GFP-expression cassette that could be used to indicate the efficiency of the infection, after 72 hours of culture, fluorescent imaging for GFP expression was conducted, and it was shown that > 90% cells were infected by lentivirus (Figure 1C). The gene silencing efficiency mediated by lentivirus was further determined using real-time quantitative PCR after culturing for 5 days, and it was shown that in both cell lines, KPNA2 expression at mRNA level was inhibited significantly, with 83% knockdown efficiency observed in HepG2 cells and 50% knockdown efficiency in SMMC-7721 cells (Figure 1D). We also examined the gene knockdown efficiency mediated by KPNA2-shRNA at the protein level in 293T cells, and the KPNA2 protein level in cells transfected with KPNA2-shRNA and cultured for 48 hours was clearly inhibited (Figure 1E).

### Blockade of cell proliferation in human hepatocellular carcinoma cells via silencing of KPNA2

**Figure 2:**
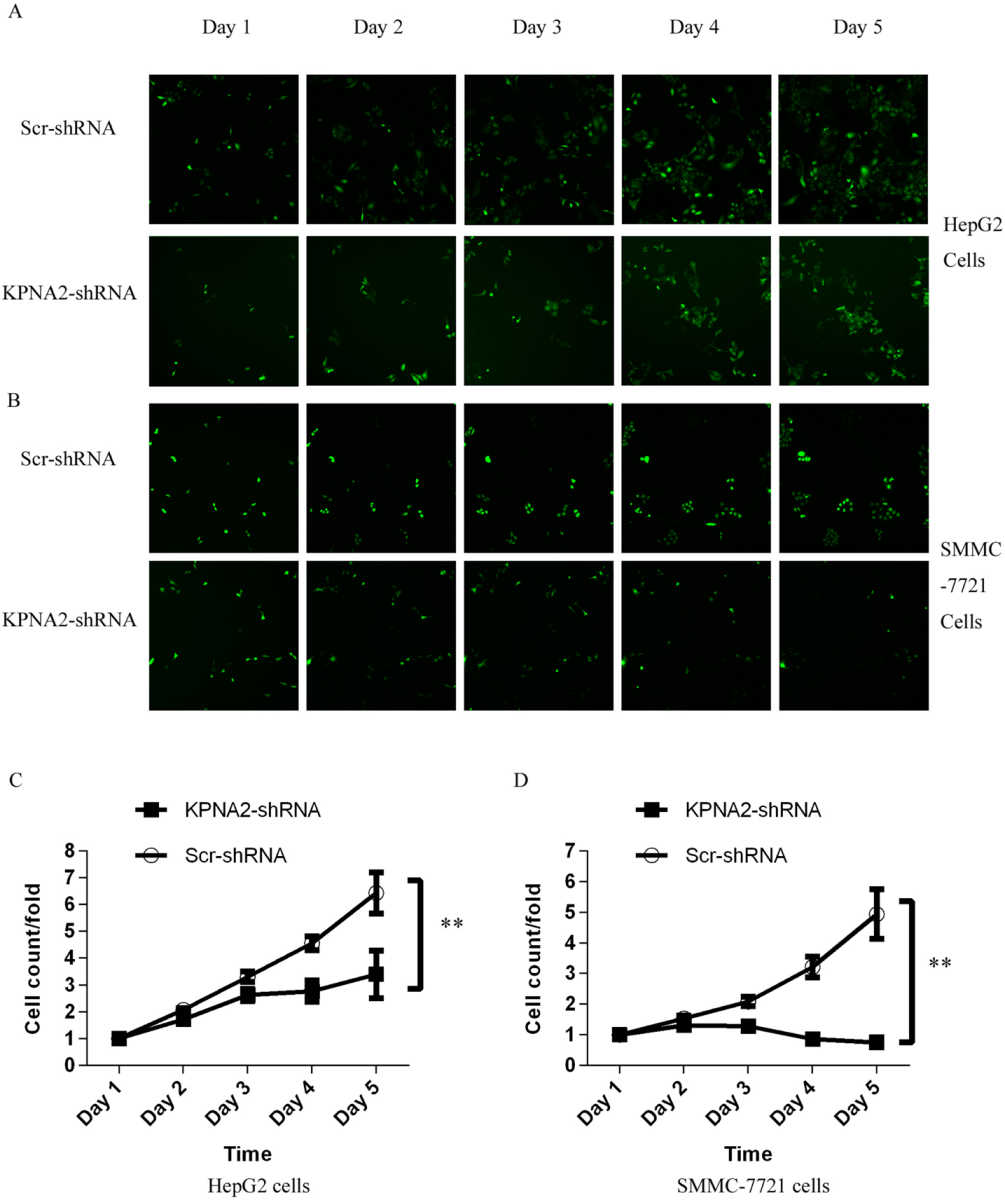
Inhibition of cell proliferation by silencing KPNA2 in human hepatocellular carcinoma cell lines HepG2 and SMMC-7721, measured via the Cellomics ArrayScan VTI (**A**–**B**) Representative microscope images of HepG2 cells (A) and SMMC-7721 cells (B) infected with lentiviruses expressing either Scr-shRNA or KPNA2-shRNA at different time points. (**C**–**D**) Proliferation profiles of HepG2 cells (C) and SMMC-7721 cells (D) infected with Scr-shRNA or KPNA2-shRNA, determined over five continuous days by the Cellomics ArrayScan VTI. The data shown here are fold changes in the number of cells and represent the mean ± SEM of three separate experiments (**p < 0.01).

To explore the involvement of KPNA2 in cell growth in human hepatocellular carcinoma, two human hepatocellular carcinoma cell lines, HepG2 and SMMC-7721, were infected with lentiviruses expressing either Scr-shRNA or KPNA2-shRNA, the efficiency of which was confirmed as described above. After 24 hours of culture, the high-content screening system Cellomics ArrayScan VTI was used to count cell numbers every day for 5 days (representative images of HepG cells and SMMC-7721 cells shown in Figure 2A and Figure 2B, respectively). Significant cell proliferation blockade was observed in both HepG2 cells and SMMC-7721 cells infected with lentiviruses expressing KPNA2-shRNA at day 4 (96 hours after lentivirus infection), and the inhibitory effect was more significant on day 5 (Figure 2C and Figure 2D show HepG and SMMC-7721 cells, respectively). Indeed, the cell number in the HepG2 line infected with lentivirus expressing KPNA2-shRNA barely increased after day 3 and even decreased, while the number of cells in the infected SMMC-7721 line barely increased after day 3.

**Figure 3:**
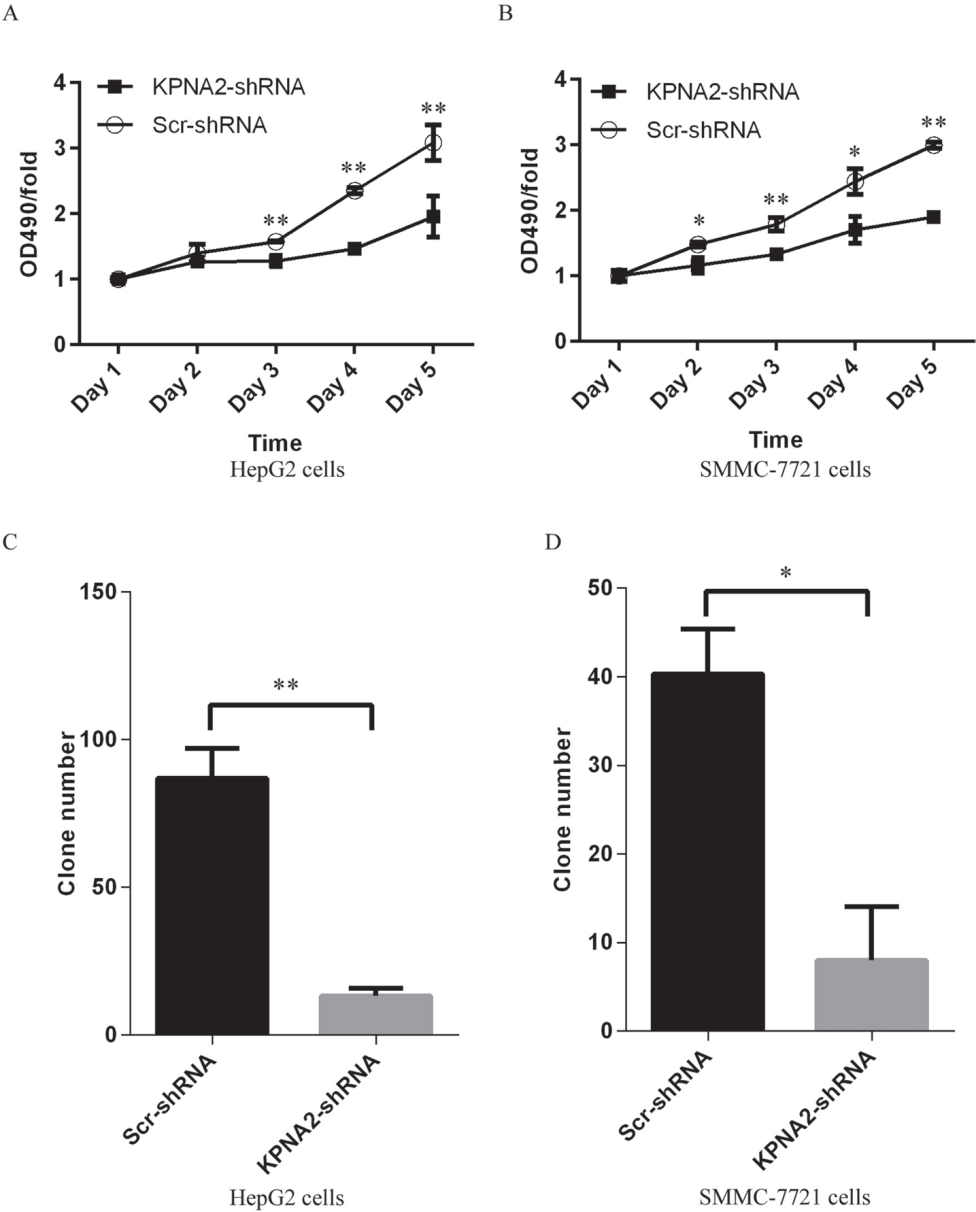
Cell proliferation blockade detected by MTT assay and impaired colony formation in human hepatocellular carcinoma cell lines HepG2 and SMMC-7721 with KPNA2 knockdown (**A**–**B**) Cell proliferation status in HepG2 cells (A) and SMMC-7721 cells (B) infected with Scr-shRNA or KPNA2-shRNA, analyzed by MTT assay over five continuous days. The data shown here are fold changes in the absorbance at OD490 and represent the mean ± SEM of three separate experiments (*p < 0.05;**p < 0.01). (**C**–**D**) Colony formation ability in HepG2 cells (C) and SMMC-7721 cells (D) infected with Scr-shRNA or KPNA2-shRNA. The data shown here are cell numbers and represent the mean ± SEM of three separate experiments (*p < 0.05;**p < 0.01).

The impact of KPNA2 knockdown on cell growth of human hepatocellular carcinoma was further examined using the MTT assay. After 24 hours of culture, cell growth profiles for the two human hepatocellular carcinoma cell lines, HepG2 and SMMC-7721, infected with either Scr-shRNA or KPNA2-shRNA were determined every day for 5 days by MTT assay. The results showed that KPNA2 knockdown inhibited cell growth significantly in both HepG cells (Figure 3A) and SMMC-7221 cells (Figure 3B). Indeed, cell growth inhibition was obvious at day 3 in HepG2 cells and at day 2 in SMMC-7721 cells infected with lentivirus expressing KPNA2-shRNA compared with cells infected with Scr-shRNA, and the inhibitory effect was sustained in the following days.

### Impaired colony formation ability in human hepatocellular carcinoma cells with KPNA2 knockdown

To investigate the impact of KPNA2 knockdown on hepatocellular carcinoma tumorigenesis, a colony formation assay was performed and showed that the colony formation ability was obviously impaired in the hepatocellular carcinoma cell lines HepG2 (Figure 3C) and SMMC-7721 (Figure 3D) infected with KPNA2-shRNA compared with cells infected with Scr-shRNA, both which were cultured for 14 days. In HepG2 cells, the average colony number was reduced from 87 to 13, while in SMMC-7721 cells, the average number was reduced from 40 to 8.

### Cell cycle arrest induced by KPNA2 knockdown in human hepatocellular carcinoma cells

**Figure 4:**
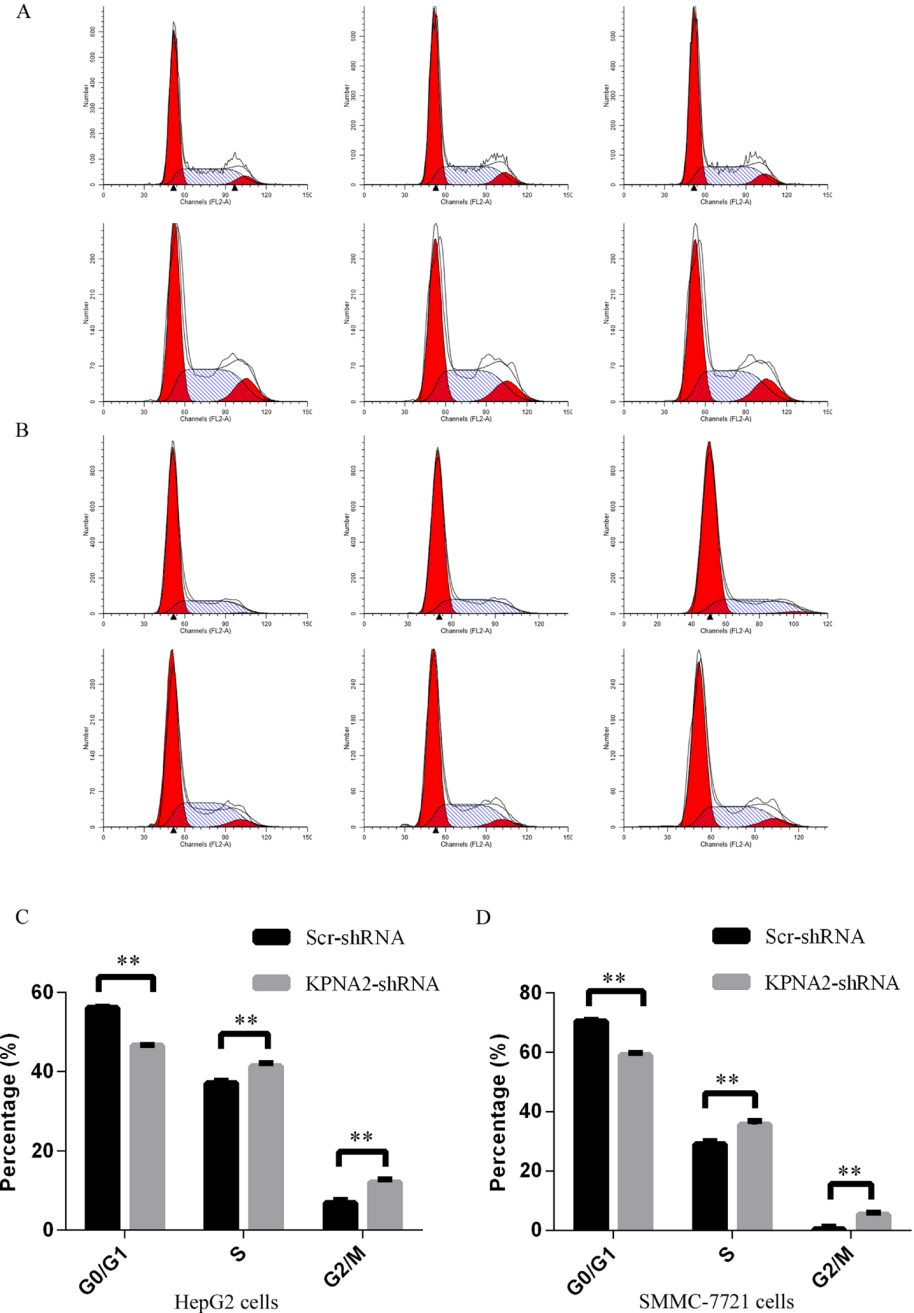
Cell cycle arrest induced in human hepatocellular carcinoma cell lines HepG2 and SMMC-7721 with KPNA2 knockdown (**A**–**B**) Representative images of cell cycle analysis for the human HepG2 cells (A) and SMMC-7721 cells (B) infected with Scr-shRNA (top) or KPNA2-shRNA (bottom). (**C**–**D**) Cell cycle distribution in HepG2 cells (C) and SMMC-7721 cells (D) infected with Scr-shRNA or KPNA2-shRNA. Both cell types infected with lentiviruses expressing either Scr-shRNA or KPNA2-shRNA were cultured for 4 days and resuspended to seed into 6-well plates for another 48 hours of culture. Cell cycle analysis was performed with flow cytometry. The graph shows the mean ± SEM of the proportion of cells in the G1 phase, S phase and G2/M phase from three separate experiments (**p < 0.01).

It was demonstrated as above that KPNA2 knockdown led to impaired cell growth and colony formation in human hepatocellular carcinoma HepG2 and SMMC-7721 cells. We further examined the cell cycle distribution in HepG2 and SMMC-7721 cells infected with lentivirus expressing either Scr-shRNA or KPNA2-shRNA, as cell cycle deregulation is an important contributor to cell growth and colony formation ability. The cell cycle distribution was determined by propidium iodide (PI) staining and flow cytometry (Figure 4A–4B), and the results showed that KPNA2 knockdown led to significant cell cycle arrest at both the S phase and the G2/M phase in both HepG2 cells (Figure 4C) and SMMC-7721 cells (Figure 4D). The percentage of cells in the G0/G1, S and G2/M phases in HepG2 cells infected with KPNA2-shRNA vs. Scr-shRNA was 46.6% vs. 56.1%, 41.4% vs. 37%, and 12% vs. 6.9%, respectively, while the percentage of infected SMMC-7721 cells in those phases was 59% vs. 70.5%, 35.6% vs. 29%, and 5.4% vs. 0.5%, respectively.

### Cellular apoptosis induced by KPNA2 knockdown in human hepatocellular carcinoma cells

**Figure 5:**
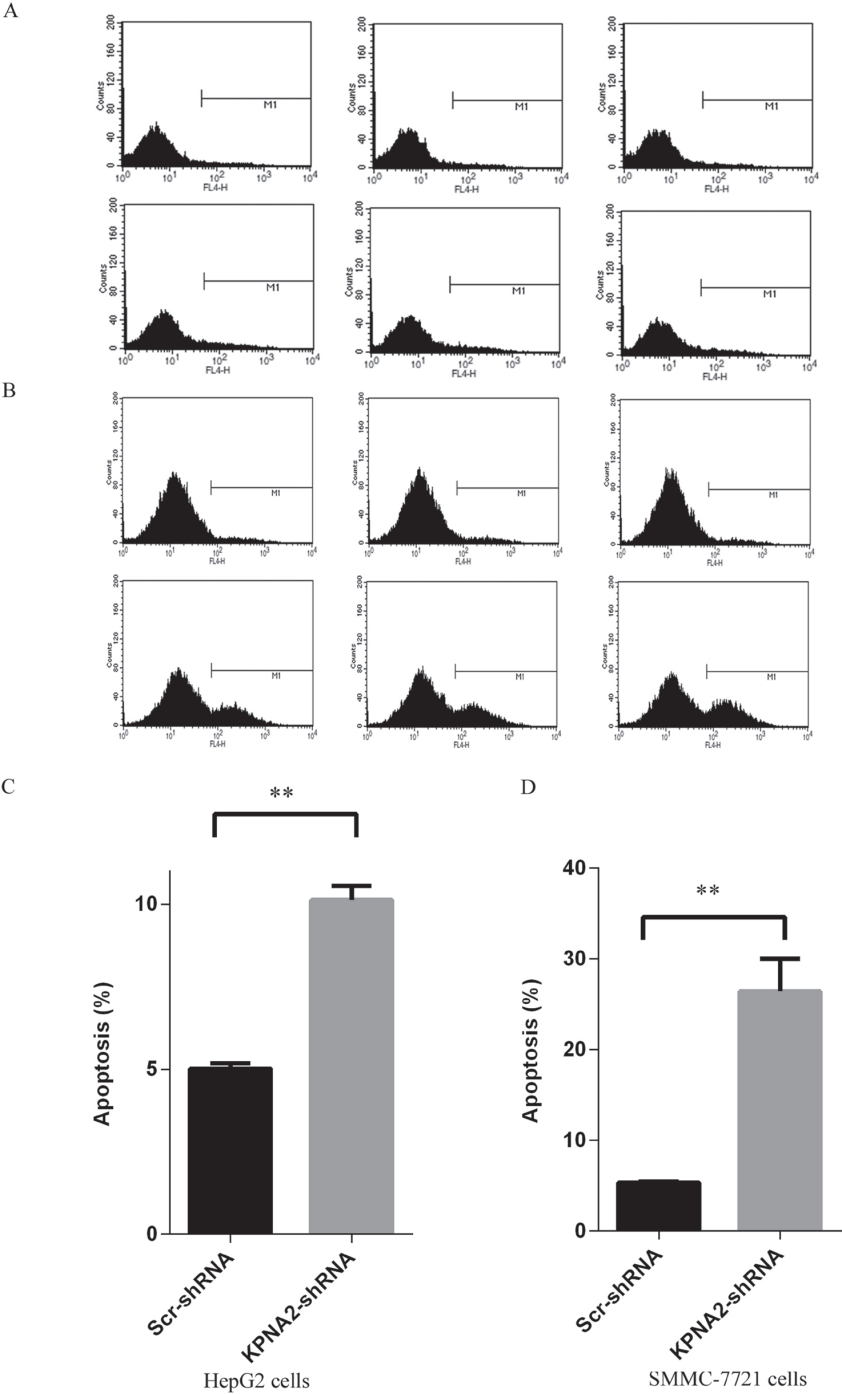
Cell apoptosis induced in human hepatocellular carcinoma cell lines HepG2 and SMMC-7721 with KPNA2 knockdown (**A**–**B**) Representative images of cell apoptosis analysis for the human HepG2 cells (A) and SMMC-7721 cells (B) infected with Scr-shRNA (top) or KPNA2-shRNA (bottom). (**C**–**D**) Cellular apoptosis in HepG2 cells (C) and SMMC-7721 cells (D) infected with Scr-shRNA or KPNA2-shRNA. Both cell types infected with lentiviruses expressing either Scr-shRNA or HSDL2-shRNA were cultured for 4 days and then resuspended for apoptosis analysis using the ANNEXIN-V assay and flow cytometry. The data shown are the mean ± SEM of apoptosis rate from three separate experiments (**p < 0.01).

Cellular apoptosis is another critical contributor to impaired cell growth and colony formation ability, so here after culturing for 4 days, the apoptosis status in human hepatocellular carcinoma cells HepG2 and SMMC-7721 infected with lentiviruses expressing either Scr-shRNA or KPNA2-shRNA was examined by the annexin V-APC assay followed by flow cytometry (Figure 5A–5B). It was shown that, for both HepG2 cells and SMMC-7721 cells, apoptosis was observed in approximately 5% cells infected with lentiviruses expressing Scr-shRNA, while the apoptosis rate reached 10% in HepG2 cells (Figure 5C) and 26% in SMMC-7721 cells (Figure 5D) infected with lentiviruses expressing KPNA2-shRNA. These results revealed that KPNA2 knockdown promoted cell apoptosis significantly in both hepatocellular carcinoma cell lines.

### Tumorigenicity of hepatocellular carcinoma cells was impaired by KPNA2 knockdown *in vivo*

**Figure 6:**
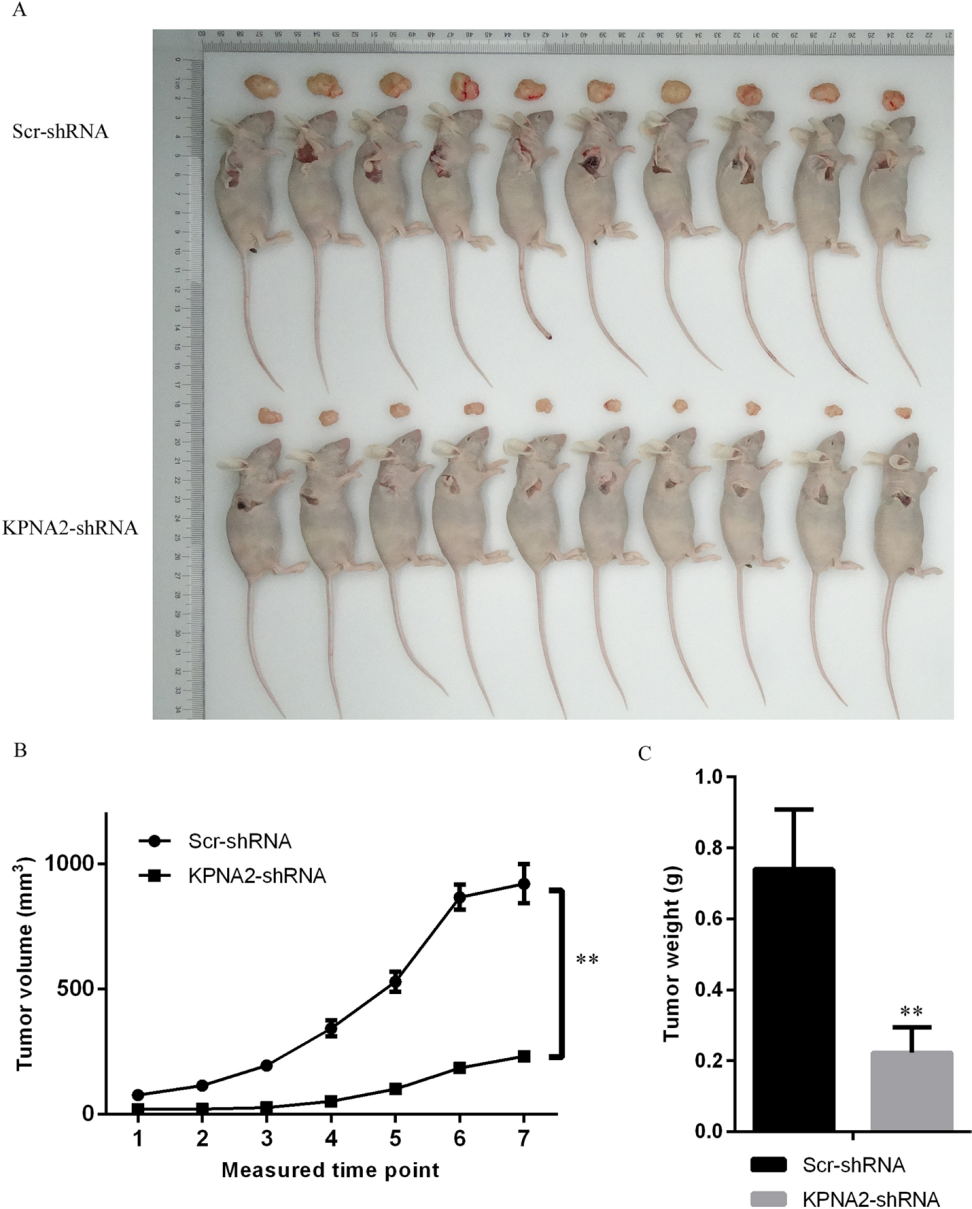
Tumorigenesis was inhibited by KPNA2 knockdown in a xenograft hepatocellular carcinoma model in nude mice (**A**) Representative images of tumor size in nude mice injected subcutaneously with SMMC-7721 hepatocellular carcinoma cells infected with either Scr-shRNA or KPNA2-shRNA. The tumor volume was examined twice each week, starting from the 10th day after tumor cell injections, for a total of 7 measurements. The mice were killed on the 29th day for tumor weight analysis. (**B**) Tumor volume examination in nude mice injected subcutaneously with SMMC-7721 hepatocellular carcinoma cells infected with either Scr-shRNA or KPNA2-shRNA. The data shown are the mean ± SEM of the tumor volume from 10 nude mice per group (**p < 0.01). (**C**) Tumor weight in nude mice injected subcutaneously with SMMC-7721 hepatocellular carcinoma cells infected with either Scr-shRNA or KPNA2-shRNA. The data shown are the mean ± SEM of tumor weight from 10 nude mice per group (**p < 0.01).

Experiments in hepatocellular cell lines HepG2 and SMMC-7721 indicated that KPNA2 is critical for the tumorigenesis of hepatocellular carcinoma cells *in vitro*, and we then wondered whether KPNA2 was important for the tumorigenicity of hepatocellular carcinoma cells *in vivo*. SMMC-7721 cells infected with lentiviruses expressing either Scr-shRNA or KPNA2-shRNA were used and injected subcutaneously into nude mice, and then the tumor volume and weight were examined. As shown in Figure 5A, the tumor size was significantly smaller in nude mice injected with cells infected with lentiviruses expressing KPNA2-shRNA than in mice injected with cells infected with lentiviruses expressing Scr-shRNA (Figure 6A). The tumor volume was smaller in nude mice injected with KPNA2-shRNA cells at all 7 time points (Figure 6B), and the tumor weight was also substantially lower in nude mice injected with KPNA2-shRNA cells (Figure 6C) than in nude mice injected with Scr-shRNA cells.

### Comprehensive analysis of gene expression changes showed regulation of key pathways in hepatocellular carcinoma cells by KPNA2

**Figure 7:**
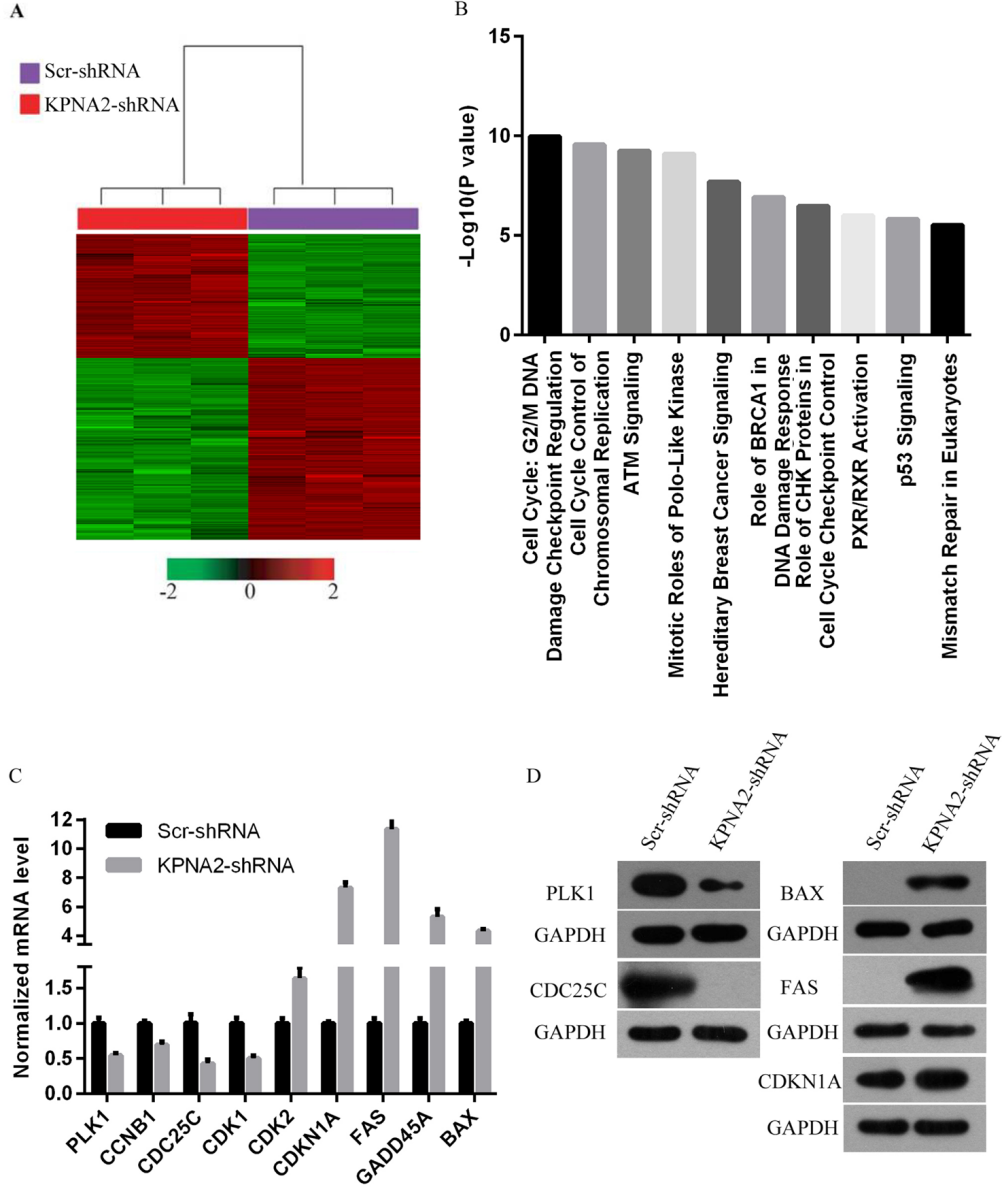
Widespread changes in gene expression and pathways essential for tumorigenesis in human hepatocellular carcinoma cell line HepG2 with KPNA2 knockdown revealed by microarray analysis (**A**) Heat map representation of 1585 genes with significant differential expressions in HepG2 human hepatocellular carcinoma cells infected with lentiviruses expressing either Scr-shRNA (purple) or KPNA2-shRNA (red) under the significance criteria of *P* < 0.05 and | fold change | > 1.5. Genes and samples are listed in rows and columns, respectively. A color scale for the normalized expression data are shown at the bottom of the microarray heat map (green represents downregulated genes, while red represents upregulated genes). (**B**) Functional pathway enrichment was analyzed using IPA. Here, the top 10 enriched pathways were shown. The statistical significance shown on the x-axis is represented by the inverse log of the *P value*. (**C**) Confirmation of microarray data using real-time quantitative PCR for selected genes PLK1, CCNB1, CDC25C, CDK1, CDK2, CDKN1A, FAS, GADD45A and BAX in HepG2 human hepatocellular carcinoma cells infected with lentiviruses expressing either Scr-shRNA or KPNA2-shRNA. The data shown here are from one of three independent experiments (*p < 0.01*) and are normalized to GAPDH. (**D**) Protein level of PLK1, CCNB1, CDC25C, CDK1, CDK2, CDKN1A, FAS, GADD45A and BAX in HepG2 human hepatocellular carcinoma cells infected with lentiviruses expressing either Scr-shRNA or KPNA2-shRNA. GAPDH was used as an internal control.

**Figure 8:**
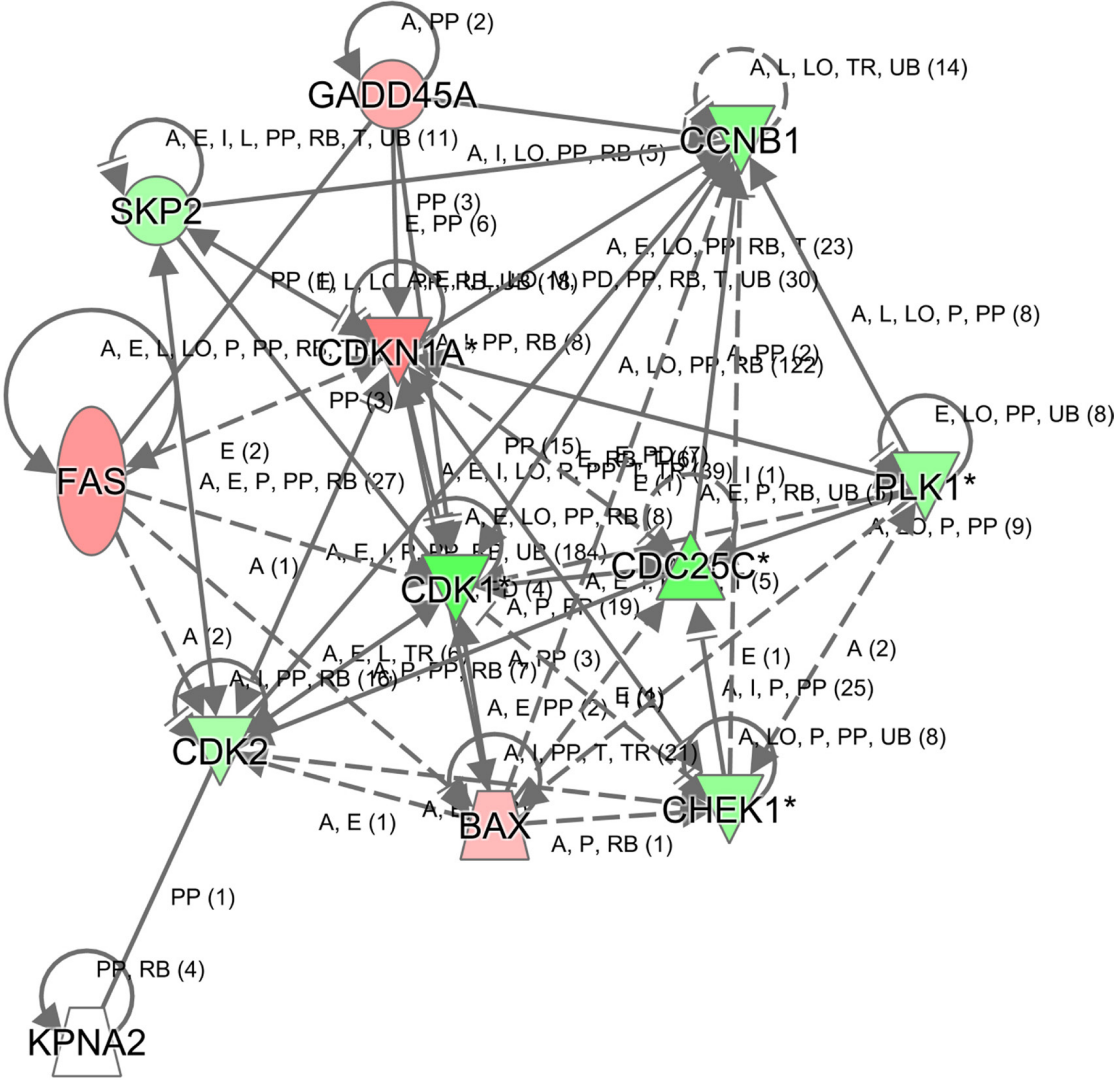
Modulation of the CDKN1A-CDK1-mediated gene interaction network in human hepatocellular carcinoma cell line HepG2 after KPNA2 knockdown In the figure, genes represented in green are downregulated, while genes in red are upregulated in HepG2 cells after KPNA2 knockdown. The solid line represents direct interactions, while the dotted line represents indirect interactions.

To further gain insights into the molecular mechanisms underlying the tumor suppression function of KPNA2 knockdown in hepatocellular carcinoma, the transcriptomes of HepG2 cells infected with lentivirus expressing either Scr-shRNA or KPNA2-shRNA were examined by a microarray platform, and a total of 1585 genes showed significantly changed expression, with *P* < 0.05 and > 1.5 absolute value of fold change (Figure 7A), including 647 upregulated genes and 938 downregulated genes. Then, the functional characteristics of these deregulated genes were analyzed using Ingenuity Pathway Analysis (IPA), and it was revealed that several critical pathways crucially involved in cancer development were deregulated by KPNA2 knockdown in HepG2 hepatocellular carcinoma cells, with the cell cycle pathway at the G2/M phase and control of chromosomal replication (S phase), the ATM signaling pathway, and the PLK kinase pathway as the top canonical pathways (Figure 7B). We further confirmed the KPNA2 knockdown-induced changes in expression of genes that are involved in the cell cycle at the G2/M phase (CDC25C, PLK1, CCNB1, GADD45A, CDKN1A), in cell cycle control of chromosomal replication (CDK2), in ATM signaling (CDC25C, CDK1, CCNB1, GADD45A, CDKN1A, CDK2), in PLK1 signaling (PLK1, CDK1, CCNB1) and in the p53 signaling pathway (BAX, FAS, GADD45A, CDKN1A, CDK2) at both the mRNA level by real-time quantitative PCR (Figure 7C) and the protein level by western blot (Figure 7D). Out of these top deregulated pathways, the cell cycle pathway at the G2/M phase and control of chromosomal replication (S phase) were involved in cell cycle regulation, while ATM signaling, PLK1 signaling and p53 signaling were critical for cell growth and survival. Indeed, the deregulated pathways enriched in differentially expressed genes induced by KPNA2 knockdown was quite in accordance with the functional effects of KPNA2 in hepatocellular carcinoma cells, which showed that KPNA2 induced cell proliferation blockade, impaired colony formation, cell cycle arrest and apoptosis. Furthermore, we performed IPA network analysis and revealed that the CDKN1A-CDK1 axis was at the core of KPNA2-mediated gene interaction networks (Figure 8), and further analysis in the future is needed.

## DISCUSSION

Aberrant, high KPNA2 expression has been observed in many cancer types, including breast cancer [16–18], melanoma [19], cervical cancer [20], esophageal cancer [21], lung cancer [22], ovarian cancer [23], prostate cancer [24], bladder cancer [25], brain cancer [26] and liver cancer [13–15]. These studies mainly focused on the correlation between the KPNA2 expression level and patient prognosis, tumor stage, or tumor malignancy, and it is well-established that KPNA2 overexpression is a marker of poor prognosis and high malignancy [10]. In hepatocellular carcinoma, it has been reported by different groups that KPNA2 expression is significantly higher in tumor regions than in the adjacent no-tumorous regions [13, 14]. These studies also revealed that KPNA2 overexpression is positively correlated with vascular invasion, tumor differentiation and tumor stage [13, 14]. Furthermore, KPNA2 has been reported by three independent groups to be a positive marker for poor prognosis and early recurrence for HCC patients [13–15], showing that both overall survival and recurrence-free survival rates were significantly lower in patients with higher KPNA2 expression. Therefore, based on the above results, it is well-established that KPNA2 is a well-defined prognostic biomarker and potential oncogene for HCC carcinogenesis. However, the roles of KPNA2 in tumorigenesis have not been explored comprehensively, and it has only been shown that cell proliferation and invasion are impaired in hepatocellular carcinoma cells *in vitro* by KPNA2 knockdown [13, 15], and *in vivo* roles of KPNA2 have remained to be determined. Furthermore, molecular mechanisms underlying KPNA2-mediated hepatocellular carcinogenesis still need in-depth investigations, as downstream signals of KPNA2 involved in cell proliferation and survival remain unknown.

Here, for the first time, we investigated KPNA2 functions systematically and in details in hepatocellular carcinogenesis *in vitro* and *in vivo* with lentiviral-mediated shRNA strategy. It was shown in two human hepatocellular carcinoma cell lines, HepG2 and SMMC-7721, that KPNA2 knockdown led to delayed cell proliferation, which was in consistent with previous report [13]. In addition, KPNA2 knockdown led to impaired colony formation ability, induced cell cycle arrest and promoted apoptosis. Furthermore, xenograft tumor mouse model experiments showed that KPNA2 knockdown could inhibit tumorigenesis *in vivo*. Taken together, these results provide reliable evidence that KPNA2 is critically involved in hepatocellular carcinoma.

In accordance with the functional results described above, our microarray analysis revealed that the corresponding pathways for dysfunction induced by KPNA2 knockdown are also enriched in KPNA2-regulated gene sets, such as the cell cycle pathways of the G2/M DNA damage checkpoint regulation and cell cycle control of chromosomal replication, which were a perfect match to the G2/M and S phase arrest observed in the human hepatocellular carcinoma cell lines HepG2 and SMMC-7721 with KPNA2 knockdown. Additionally, ATM signaling and mitotic roles of PLKs enriched in KPNA2-associated gene sets were also responsible for the cell cycle arrest observed in cells treated with KPNA2 knockdown [27, 28]. Furthermore, ATM signaling and p53 signaling were also involved in the regulation of cell survival, so the dysfunctions of these two signaling pathways induced by KPNA2 knockdown were at least partially responsible for the elevated apoptosis in HepG2 and SMMC-7721 cells treated with KPNA2-shRNA [27, 29–31]. Indeed, it has been demonstrated that KPNA2 induced p53 expression and essential for the regulation of p53 nuclear translocation, so it is quite possible that KPNA2 promoted hepatocellular carcinogenesis partially through p53 signaling [32, 33].

In addition to pathway enrichment analyzed for KPNA2-associated gene sets, the expression of several critical genes involved in cell cycle regulation and cell survival was confirmed by real-time quantitative PCR and western blot. Out of these genes, the expression of PLK-1, responsible for the G2/M transition and cell survival [34], was decreased in HepG2 cells and SMMC-7721 cells treated with KPNA2-shRNA. It is well-known that, in the G2/M transition process, PLK-1 induces CDC25 activation to promote CDK1 activation [35], and here we found that all three of these proteins were inhibited by KPNA2 knockdown in hepatocellular carcinoma cells, suggesting that KPNA2 silencing-induced G2/M phase arrest could occur through the PLK1-CDC25-CDK1 axis [36]. In the cellular apoptotic process, PLK1 was able to inhibit the expression of two apoptosis-related proteins, CDKN1A and BAX, indicating that KPNA2 silencing-induced cellular apoptosis can be partially attributed to the PLK1-CDKN1A/BAX axis [34]. In addition, FAS protein, upregulated in HepG2 cells and SMMC-7721 cells treated with KPNA2-shRNA, is an inducer of cellular apoptosis [37], and FAS-mediated apoptosis can be blocked by CDK1 [38]. Thus, it is quite possible that KPNA2 knockdown interfered with the normal functions of multiple cross-linked pathways essential for cell proliferation and survival, leading to the obvious cell proliferation blockade and elevated apoptosis in hepatocellular carcinoma cells. In consideration of the functions of KPNA2 in nucleocytoplasmic transport, we speculated that nucleocytoplasmic transport of transcriptional regulators essential for the expression of factors in cell proliferation and survival pathways might occur through KPNA2 and that aberrant expression of KPNA2 leads to abnormal localization and impaired functions of these regulators, which further promotes hepatocellular carcinogenesis. However, this speculation needs experimental verification in the future to expand our understanding of the functions of KPNA2 in different cancers.

In summary, with *in vitro* and *in vivo* experiments, causal relationship between KPNA2 upregulation and hepatocellular carcinoma progression was more convincing in this study. Furthermore, microarray analysis unraveled potential downstream targets of KPNA2, providing insights for mechanisms underlying KPNA2-associated hepatocellular carcinogenesis. These results paves the way for further in-depth analysis of KPNA2's roles in other cancer types. Indeed, network analysis for KPNA2-regulated gene sets clarified the signals underlying different aspects of cellular processes, such as cell cycle arrest at the G2/M phase or cellular apoptosis in hepatocellular carcinoma cells with KPNA2 knockdown, and this suggested that KPNA2 is a multifunctional factor involved in diverse aspects of tumorigenesis and tumor progression. In the future, it will be necessary and valuable to explore the therapeutic potential of targeting KPNA2 for cancer treatment.

## MATERIALS AND METHODS

### Culture of hepatocellular carcinoma cell lines

The human embryonic kidney cell line 293T cells and four human hepatocellular carcinoma cell lines, Hep3B, Huh-7, HepG2 and SMMC-7721, were obtained from the Cell Bank of the Type Culture Collection of the Chinese Academy of Sciences (Shanghai, China). 293T cells and Hep3B, Huh-7 and HepG2 cells were cultured in Dulbecco's Modified Eagle's medium (DMEM) containing 10% fetal bovine serum (FBS) and 1% antibiotics while SMMC-7721 cells were cultured in RPMI 1640 medium containing 10% FBS and 1% antibiotics. Both cells were incubated in a 5% CO_2_ incubator at 37°C.

### shRNA design and lentivirus preparation

To achieve a specific and efficient gene silencing effect, short-hairpin RNA (shRNA) specifically targeting the human *KPNA2* gene (sequence: 5′-CTACCTCTGAAGGCTACACTT-3′) was designed, and related dsDNA oligonucleotides were synthesized by Genechem (Shanghai, China). DNA oligonucleotides were annealed and inserted into the lentiviral vector pGCSIL-GFP (Genechem, Shanghai, China), and lentiviruses expressing scrambed shRNA (Scr-shRNA) (negative control, sequence: 5′-TTCTCCGAACGTGTCACGT-3′) or shRNA specifically targeting the human *KPNA2* gene (KPNA2-shRNA) was prepared using the Lentivector Expression System (Genechem, Shanghai, China) for subsequent experiments.

### shRNA knockdown efficiency assay

To examine the *KPNA2* knockdown efficiency by the designed shRNA for subsequent applications, 293T cells were infected with a Flag-KPNA2-overexpressing plasmid, and Scr-shRNA or KPNA2-shRNA using Lipofectamine 2000 (Invitrogen, Carlsbad, CA, USA). After 48 hours of culture, total proteins were isolated to examine the expression level of Flag-KPNA2 protein with GAPDH protein as internal control.

### Infection of human hepatocellular carcinoma cells with lentiviruses

To investigate the roles of KPNA2 in hepatocellular carcinoma, human hepatocellular carcinoma cells HepG2 and SMMC-7721 were infected with lentiviruses expressing either Scr-shRNA or KPNA2-shRNA. In brief, cells were seeded into 6-, 12- or 24-well plates, depending on experimental requirements. After reaching an appropriate density, cells were infected with lentiviruses expressing either Scr-shRNA or KPNA2-shRNA and cultured for another 2–5 days. The lentiviral infection efficiency was determined by the percentage of cells that was GFP-positive cells, examined using a fluorescence microscope and then used for subsequent experiments.

### Total RNA isolation, cDNA synthesis and real-time quantitative PCR

HepG2 cells and SMMC-7721 cells infected with lentiviruses expressing either Scr-shRNA or KPNA2-shRNA were cultured for 5 days, and then total RNA was extracted for cDNA synthesis and real-time quantitative PCR. In brief, total RNA was extracted with TRIzol reagent (Invitrogen, Carlsbad, CA, USA) according to the manufacturer's instructions. After RNA quantification using a NanoDrop (Thermo, Rockford, IL, MA, USA), cDNA fragments were synthesized using M-MLV Reverse Transcriptase (Promega, Madison, Wisconsin, USA) and Oligo dT primers (Sangon, Shanghai, China) according to the manufacturers’ instructions. The expression level of HSDL2 and other downstream targets identified in the microarray assay was determined using real-time quantitative PCR using a SYBR master mixture (Takara Biotechnology, Dalian, China) on a TP800 real-time PCR machine (Takara Biotechnology, Dalian, China). Primers used in this study were designed with Beacon Designer 2 and synthesized by Genechem (Shanghai, China). The sequences of the primers used are as follows:

GAPDH forward: 5′-TGACTTCAACAGCGACA CCCA-3′; GAPDH reverse: 5′-CACCCTGTTGCTGTA GCCAAA-3′; KPNA2 forward: 5′- TGTGGTAGATG GAGGTGC-3′;KPNA2 reverse: 5′- GAGCCAACAG TGGGTCA-3′; CDC25C forward: 5′- ATGACAATGGAA ACTTGGTGGAC-3′;CDC25C reverse: 5′- GGAGCGAT ATAGGCCACTTCTG-3′; CDK1 forward: 5′-GGATGTGC TTATGCAGGATTCC-3′;CDK1 reverse: 5′- CATGTA CTGACCAGGAGGGATAG-3′; CDK2 forward: 5′- CTGG ACACTGAGACTGAGG-3′;CDK2 reverse: 5′- GAGG ACCCGATGAGAATGG-3′; PLK1 forward: 5′- AGG CAAGAGGAGGCTGAG-3′;PLK1 reverse: 5′- GGAT GAGGCGTGTTGAGTC-3′; CCNB1 forward: 5′- CTAAGATTGGAGAGGTTGATGTC-3′;CCNB1 reverse: 5′- GGTAATGTTGTAGAGTTGGTGTC-3′; CDKN1A forward: 5′- GGGACAGCAGAGGAAGACC-3′;CD KN1A reverse: 5′- GACTAAGGCAGAAGATG TAGAGC-3′; FAS forward: 5′- CTTCTTTTGCCAAT TCCAC-3′;FAS reverse: 5′- CAGATAAATTTATTG CCACTG-3′; GADD45A forward: 5′- GAGAGCA GAAGACCGAAAGG-3′;GADD45A reverse: 5′- CAGC AGGCACAACACCAC-3′; BAX forward: 5′- TGCTT CAGGGTTTCATCCA-3′;BAX reverse: 5′-GGC CTTGAGCACCAGTTT-3′;

### Western blotting for protein expression analysis

HepG2 cells and SMMC-7721 cells infected with lentiviruses expressing either Scr-shRNA or KPNA2-shRNA and 293T cells were cultured for 48 hours, and then total protein was extracted as follows: cells were collected and cells were lysed with lysis buffer (100 mM Tris-HCl, pH = 7.41; 0.15 M NaCl; 5 mM EDTA, pH = 8.0; 1% Triton-X100; 5 mM DTT; 0.1 mM PMSF) to isolate total protein samples. The total protein concentrations were quantified using a BCA Protein Assay Kit (Pierce, Rockford, IL, USA). Then, 5X loading buffer was added to 20 μg of total protein for western blot analysis. Protein samples were separated using appropriate SDS-PAGE gels and transferred to PVDF membranes (Amersham Biosciences, Pollards Wood, UK). For blocking, membranes were incubated in 5% milk in TBST at room temperature for 1 hour, and then primary antibodies were added for overnight incubation at 4°C. The primary antibodies used here are as follows: rabbit anti-CDKN1A, Abcam, ab7960 (1:500); rabbit anti-PLK1, Cell Signaling, #4513 (1:1000); rabbit anti-FAS, Abcam, ab82419 (1:1000); rabbit anti-BAX, Abcam, ab7977 (1:1000); rabbit anti-CDC25C, Cell Signaling, #4688 (1:1000); mouse anti-Flag, Sigma, F1804 (1:1000); and mouse anti-GAPDH, Santa-Cruz, sc-32233 (1:2000). After washing three times with 1X TBST buffer, a secondary antibody coupled to HRP from Santa Cruz was added, and immunoactivity was detected using an ECL-Plus kit (Amersham Biosciences, Pollards Wood, UK).

### Cellomics ArrayScan VTI assay for cell proliferation analysis

HepG2 cells and SMMC-7721 cells infected with lentiviruses expressing either Scr-shRNA or KPNA2-shRNA were collected in the logarithmic growth phase and seeded in triplicate into 96-well plates at a final density of 2000 cells/well. After another 24 hours of culture, cell numbers were autonomously quantified using the Cellomics ArrayScan VTI (Thermo, Rockford, IL, MA, USA) with a 488 nm laser once a day for five continuous days. Then, cell growth curves were produced for each condition.

### MTT assay

The MTT assay was used to determine cell growth status as follows: HepG2 cells and SMMC-7721 cells infected with lentiviruses expressing either Scr-shRNA or KPNA2-shRNA were collected in the logarithmic phase and seeded into 96-well plates in triplicate at a final density of 2000 cells/well. Then, cell growth profiles were determined each day for 5 continuous days. In brief, 20 μL of MTT solution (5 mg/mL) was added into each well and incubated with the cells for 4 hours. The culture medium was removed totally, and 150 μL of DMSO was added to dissolve the formazan. Absorbance at 490 and 570 nm was quantified using a microplate reader after shaking the plate constantly for 5–10 minutes.

### Colony formation assay

HepG2 cells and SMMC-7721 cells infected with lentiviruses expressing either Scr-shRNA or KPNA2-shRNA were cultured for 48 h to reach the logarithmic growth phase. Cells were then collected and counted with a hemocytometer. To analyze the colony formation ability, cells were plated in triplicate into six-well plates at a density of 800 cells/well and cultured for 14 days. After fixation with paraformaldehyde for 30–60 min, cells were stained with GIEMSA for 20 min. Images were then obtained with micropublisher 3.3RTV (Olympus) after the cells were washed several times with ddH_2_O. Cell colonies were counted and quantified using ImageJ.

### Cell cycle distribution analysis

Flow cytometry was used for cell cycle distribution analysis as previously described [39]. In brief, HepG2 cells and SMMC-7721 cells infected with lentiviruses expressing either Scr-shRNA or KPNA2-shRNA were cultured for 96 hours to prepare cell suspensions. After the cells were counted, they were plated into 6-cm dishes and cultured for 48 hours to reach approximately 80% coverage. Then, cells were washed, collected and fixed using cold 70% alcohol for at least 1 hour. Cells were mixed with PI buffer containing 40 × PI stock (2 mg/ml), 100 × RNase stock (10 mg/ml) and 1 × PBS buffer at a dilution of 25:10:1000 after washing with PBS buffer. Then, cell cycle distribution was examined using a FACSCalibur (Becton-Dickinson, San Jose, CA, USA) with at least 1 × 10^6^ cells per sample. At least three independent experiments were performed for cell cycle distribution analysis.

### Cellular apoptosis analysis with the annexin V-APC assay

The Annexin V-APC apoptosis detection kit (eBioscience, San Diego, CA, USA) was chosen for apoptosis analysis, and experiments were performed according to the manufacturer's instructions. In brief, HepG2 cells and SMMC-7721 cells infected with lentiviruses expressing either Scr-shRNA or KPNA2-shRNA were cultured for 4 days. Then, cells were collected and washed with PBS to prepare cell suspensions with staining buffer at a final density of 1 × 10^6^−1 ×10^7^/ml. The 100-μl cell suspensions were mixed with 5 μl annexin V-APC and incubated at room temperature for 10–15 min. Cell apoptosis was then determined using a FACSCalibur (Becton-Dickinson, San Jose, CA, USA).

### Tumorigenicity in nude mice

Experiments on nude mice for tumorigenesis analysis were approved by the Institutional Animal Care and Use Committee. All nude mice were fed strictly according to the institution's guidelines. A hepatocellular carcinoma xenograft model in nude mice was established as follows: SMMC-7721 cells infected with lentiviruses expressing either Scr-shRNA or KPNA2-shRNA were cultured for 48 h to reach the logarithmic growth phase. Cells were then collected and counted with a hemocytometer and resuspended in PBS at a density of 2 × 10^7^ cells/ml, and nude mice were subcutaneously injected with approximately 4 × 10^6^ cells. Two groups were divided according to the lentiviruses used: the control group was injected with SMMC-7721 cells infected with lentiviruses expressing Scr-shRNA and knockdown group injected with SMMC-7721 cells infected with lentiviruses expressing KPNA2-shRNA. Then, nude mice were fed, and the tumor diameter was measured two times each week, starting from the 10th day, with a frequency of two times a week, for a total of 7 measurements. The mice were killed on the 29th day for tumor weight analysis.

### Gene expression microarray analysis

Total RNA was extracted from HepG2 cells infected with lentiviruses expressing either Scr-shRNA (*n* = 3) or KPNA2-shRNA (*n* = 3) using Trizol reagents. Then, RNA quantity and quality were determined using a NanoDrop 2000 and an Agilent Bioanalyzer 2100. Gene expression analysis was determined using the Affymetrix GeneChip PrimeView Human Gene Expression Array according to the manufacturer's instructions. Briefly, the GeneChip 3′ IVT Expression Kit was used for reverse transcription, double-stranded DNA template conversion, *in vitro* transcription for aRNA synthesis and labeling. The GeneChip Hybridization Wash and Stain Kit was used for microarray hybridization, washing and staining. Raw data were collected using the GeneChip Scanner 3000 for array scanning. Genes with significantly differential expression between HepG2 cells infected with lentiviruses expressing Scr-shRNA (*n* = 3) and cells infected with lentiviruses expressing KPNA2-shRNA (*n* = 3) were selected based on the following criteria: *P value* < 0.05 and absolute fold change > 2. Pathway enrichment analysis was performed for all significant differential genes based on Ingenuity Pathway Analysis (IPA).

### Statistical analysis

GraphPad Prism 6 software was used for statistical analysis, and all of the experiments were repeated at least three times. Data are presented as the mean ± SEM of three independent experiments. Student's two-tailed *t-test* was used to quantify significant differences, and values of *P* < 0.05 were considered statistically significant.
